# Development and Application of EST-SSR Markers to Assess Genetic Diversity and Structure of *Eleutherococcus senticosus* for Conservation and Breeding

**DOI:** 10.3390/plants15060860

**Published:** 2026-03-10

**Authors:** Shikai Zhang, Luwei Ding, Cheruiyot Evans, Eliamani Singo, Jiawei Wu, Guanzheng Qu, Tuya Siqin, Xuefeng Han, Shunjie Zhang, Xiangling You

**Affiliations:** 1 Key Laboratory of Saline-Alkali Vegetation Ecology Restoration, Ministry of Education, Northeast Forestry University, Harbin 150040, China; 2 State Key Laboratory of Tree Genetics and Breeding, School of Forestry, Northeast Forestry University, Harbin 150040, China; 3 Kenya Forestry Research Institute (KEFRI), Nairobi P.O. Box 20412-00200, Kenya; 4 Heilongjiang Institute of Atomic Energy, Harbin 150081, China; 5 Forestry and Grassland Administration of Tangyuan County, Jiamusi 154799, China; 6 Research Center for Medicinal Resources, Mudanjiang Branch, Heilongjiang Academy of Forestry, Mudanjiang 157006, China

**Keywords:** natural populations, genetic diversity, genetic structure, genetic differentiation

## Abstract

*Eleutherococcus senticosus*, a medicinally important woody plant, is widely used in pharmaceuticals and functional foods due to its bioactive compounds. Its wild populations are facing severe threats due to over-harvesting. To inform scientific conservation and sustainable utilization strategies, this study aimed to comprehensively assess its genetic background. We developed 13 highly polymorphic expressed sequence tag-simple sequence repeat (EST-SSR) markers from full-length transcriptome data, with an average polymorphism information content (*PIC*) of 0.52. Using these markers, we systematically evaluated the genetic diversity of 405 individuals from 22 natural populations across Northeast China. The results indicate that *E. senticosus* maintains moderate genetic diversity at the species level (mean expected heterozygosity *He* = 0.43), but substantial variation exists among populations. The Linjiang population showed the highest diversity (*He* = 0.58), whereas peripheral populations such as Tonghua (*He* = 0.31) and Huinan (*He* = 0.32) exhibited lower diversity. Analysis of molecular variance (AMOVA) revealed that genetic variation primarily resided within populations (66.3%), but moderate differentiation among populations was also detected (*Fst* = 0.21). Both structure analysis and clustering consistently divided all populations into two major genetic lineages. Frequent gene flow (e.g., *Nm* > 10 between Raohe and Hulin) and high genetic homogeneity were observed among populations in the core distribution area (e.g., Raohe, Jixi, Hulin), whereas several peripheral populations displayed significant genetic distinctiveness and isolation. This study provides the first macro-scale insight into the population genetic structure of *E. senticosus*, offering crucial molecular tools and a scientific basis for in situ and ex situ conservation, core collection establishment, and future genetic improvement of this species.

## 1. Introduction

*Eleutherococcus senticosus* is an important medicinal species within the temperate forest ecosystems of East Asia [1]. Its natural populations are widely distributed across Northeast China, the Russian Far East, and the Korean Peninsula [2]. The roots, stems, and leaves of *E. senticosus* are rich in various bioactive compounds [3,4,5], including triterpenoids [6], flavonoids, polysaccharides [7], and syringin [8], which have demonstrated significant immunomodulatory [4,9], anti-fatigue, antioxidant [10], and neuroprotective pharmacological activities [11,12,13]. Due to its considerable ecological value and economic potential, the species is increasingly utilized in pharmaceuticals, functional foods, and health products driven by the growing wellness industry [1,14]. Widespread commercial recognition of *E. senticosus* was gained in the mid-to-late 20th century. Intensified and largely unregulated harvesting of wild roots was triggered by this surge in demand from the 1980s onward. However, the sustainable utilization of this valuable resource faces severe challenges. Persistent and rising market demand, combined with insufficient regulatory oversight, has driven commercial harvesting of *E. senticosus* to rely almost exclusively on the destructive collection of wild roots and rhizomes. This practice has precipitated a sharp decline in natural population stocks. Concurrent habitat degradation due to expanding human activity has further compromised the species’ survival, rendering this valuable medicinal plant highly endangered.

*E. senticosus* belongs to a genus comprising several medicinally relevant species, such as *E. sessiliflorus* and *E. gracilistylus*, which also contain bioactive compounds but remain genetically understudied. Within this genus, *E. senticosus* is a perennial deciduous shrub characterized by slow growth, poor natural regeneration, and self-incompatibility [15]. Consequently, over-harvesting and habitat destruction readily lead to local population extinction and can trigger severe erosion of intraspecific genetic diversity, loss of rare alleles, and degradation of population genetic structure. Genetic diversity is fundamental for a species to adapt to environmental changes and maintain long-term evolutionary potential [16,17]. Its decline in *E. senticosus* would directly impair the species’ capacity to cope with stressors such as climate change and disease, ultimately threatening the sustainability of its use as a valuable medicinal resource.

Therefore, a systematic genetic assessment of *E. senticosus* germplasm is essential for its conservation, sustainable utilization, and genetic improvement. Traditional germplasm evaluation largely relies on morphological and physiological traits [18,19], which, while practical, are often influenced by environmental factors and seldom reveal the underlying genetic differences among populations. Advances in molecular biology and high-throughput sequencing have established DNA-based molecular markers as powerful tools for analyzing genetic diversity and deciphering population structure [20,21]. Among various molecular markers, expressed sequence tag-simple sequence repeat (EST-SSR) markers, derived from transcribed coding regions, offer advantages such as codominant inheritance, high polymorphism, reproducibility, and relatively low development costs from transcriptomic data, and potential functional relevance [22,23]. While genomic SSRs and high-throughput SNP arrays (e.g., from GBS) can offer higher genome coverage and polymorphism, they often entail greater initial cost and computational complexity for non-model species. For the objectives of this study—establishing a reliable marker set and conducting a macro-scale population genetic assessment of *E. senticosus*—EST-SSRs provide an optimal balance between information content, cost, and technical accessibility. They have been effectively applied in genetic diversity analyses, core germplasms construction, and population genetic structure analyses of multiple timber species—including *Juglans mandshurica* [24], *Pinus koraiensis* [25], and *Eucalyptus cloeziana* [26]—as well as some medicinal plants such as *Panax ginseng* [27], *Panax notoginseng* [28], *Pinellia ternata* [29] and more. These applications provide direct evidence for informing targeted conservation strategies and germplasm improvement.

**Figure 1:**
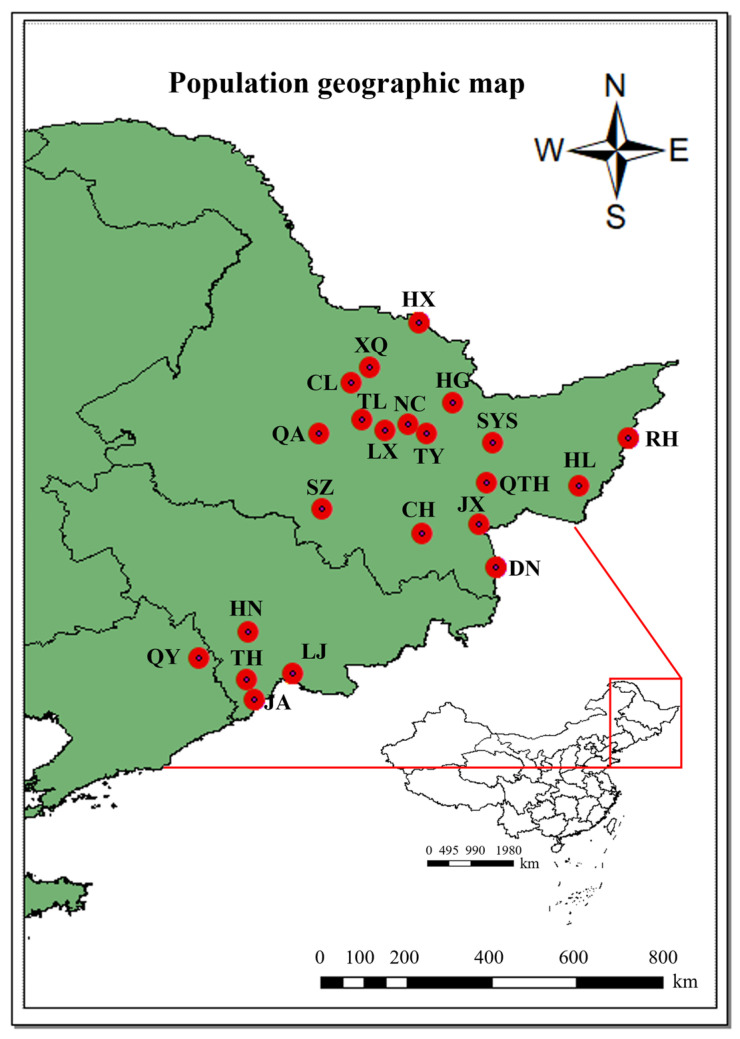
Geographical distribution of the 22 sampled *E. senticosus* populations in Northeast China.

Current research on the genetic diversity of *E. senticosus* remains relatively limited, with existing studies focusing predominantly on chemical composition [30], pharmacological activity [31], and functional analysis of key genes [32]. Few reports have employed molecular markers to examine genetic diversity at the provenance level [33,34,35]. To address this knowledge gap and to establish an efficient and reliable molecular toolkit for resource conservation and breeding, this study collected 405 germplasm samples from 22 populations across the forested regions of Northeast China (Figure 1). While *E. senticosus* has a broader trans-national distribution, this study focuses on its natural populations within Northeast China, which constitutes a major and ecologically diverse part of its native range and is currently under the most intense harvesting pressure. Assessing the genetic resources within this critical region is an urgent priority for formulating national conservation strategies. Based on transcriptome sequencing data of *E. senticosus*, we developed polymorphic EST-SSR primers and applied these markers to analyze the genetic diversity, population structure, and differentiation among natural populations in northeastern China. The extent of gene flow among geographical groups was also assessed. This work not only provides a molecular basis for the ecological conservation, core collection assembly, and genetic improvement of this important forest medicinal resource, but also establishes a genetic foundation for subsequent trait association analysis and marker-assisted selection breeding.

## 2. Results

### 2.1. Identification of EST-SSR Loci

**Figure 2:**
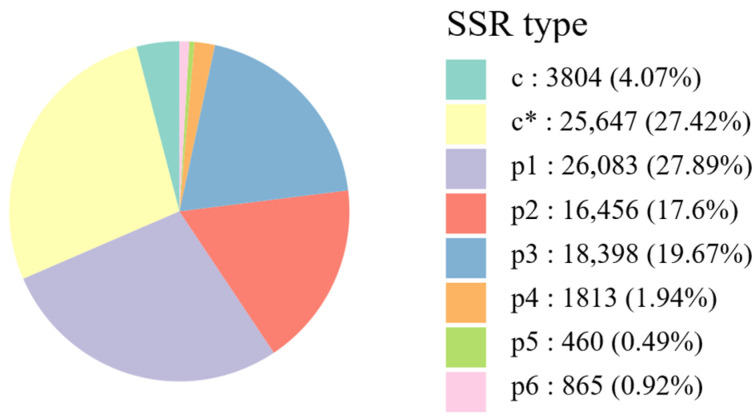
Distribution and proportions of different motif types identified in *E. senticosus* EST-SSRs. * indicates composite SSRs (c*-type).

A total of 93,526 EST-SSR loci were identified from the full-length transcriptome data of *E. senticosus*. These loci were classified and counted by repeat-unit type (Figure 2). Mononucleotide repeats (p1-type) were the most abundant (26,083 loci), accounting for 27.89% of all SSR loci. Among these, repeats of 10 units were the most frequent (17,989 loci, 19.25% of total SSRs). Composite SSRs (c*-type) represented the second most common category (25,647 loci, 27.42%), followed by dinucleotide (p2-type; 16,456 loci, 17.60%) and trinucleotide (p3-type; 18,398 loci, 19.67%) repeats. Tetranucleotide (p4-type), pentanucleotide (p5-type), and hexanucleotide (p6-type) repeats were relatively scarce, constituting 1813 (1.94%), 460 (0.49%), and 865 (0.92%) loci, respectively. In addition, 3804 perfect compound SSRs (c-type) were detected, representing 4.07% of the total.

Overall, mononucleotide, composite, dinucleotide, and trinucleotide repeats collectively constituted 92.48% of all SSR loci. Regarding repeat-number distribution, SSRs with five and six repeat units were predominant aside from mononucleotide and composite repeats, with counts of 12,950 (13.85%) and 13,155 (14.07%), respectively (Table 1). These results indicate that the transcriptome of *E. senticosus* harbors abundant SSR loci with high potential polymorphism, providing a solid foundation for developing polymorphic EST-SSR markers and subsequent population genetic studies.

### 2.2. Screening of Polymorphic EST-SSR Primers

Following initial screening of the developed EST-SSR primers for *E. senticosus*, a total of 96 primer pairs were randomly selected and synthesized. Subsequent screening using DNA samples from the 22 populations yielded 13 primer pairs that exhibited stable amplification, clear banding patterns, and high polymorphism, which were used for subsequent genetic diversity analyses (Table 2). Across all tested materials, the 13 EST-SSR loci collectively detected 86 alleles. The total number of alleles per locus (*Na*) ranged from 3 (Ese-20) to 13 (Ese-22), with a mean of 6.62. The effective number of alleles (*Ne*) ranged from 1.44 (Ese-74) to 3.01 (Ese-22), averaging 1.99. Shannon’s information index (*I*) varied from 0.40 (Ese-74) to 1.25 (Ese-22), with a mean of 0.73. Observed heterozygosity (*Ho*) ranged from 0.11 (Ese-24) to 0.95 (Ese-22), averaging 0.46, while expected heterozygosity (*He*) varied from 0.24 (Ese-74) to 0.66 (Ese-22), with a mean of 0.43. Polymorphism information content (*PIC*) values ranged from 0.27 (Ese-74) to 0.77 (Ese-22), averaging 0.52, indicating moderate to high polymorphism across the selected loci.

Analyses of population genetic structure parameters showed that the total fixation index (*Fit*) ranged from −0.33 (Ese-14) to 0.82 (Ese-24), with a mean of 0.18. The inbreeding coefficient within subpopulations (*Fis*) varied from −0.81 (Ese-55) to 0.74 (Ese-24), averaging −0.06. Genetic differentiation among populations (*Fst*) ranged between 0.07 (Ese-20) and 0.51 (Ese-65), with a mean of 0.24, indicating moderate genetic differentiation among populations. Correspondingly, estimated gene flow (*Nm*) ranged from 0.24 (Ese-65) to 3.16 (Ese-20), with an average of 1.14 across loci.

### 2.3. Genetic Diversity Analysis of E. senticosus Populations

Genetic diversity was assessed for the 22 geographic populations (405 samples) of *E. senticosus* using the 13 selected polymorphic EST-SSR primers (Table 3). The genetic diversity parameters varied among populations. The mean number of alleles (*Na*) ranged from 2.15 (HN, TH) to 3.92 (LJ), with a mean of 2.84. The effective number of alleles (*Ne*) varied from 1.57 (TH) to 2.52 (LJ), averaging 1.99. Shannon’s information index (*I*) ranged from 0.50 (TH, HN) to 1.03 (LJ), with a mean of 0.73. Observed heterozygosity (*Ho*) ranged from 0.25 (TH) to 0.63 (JX), averaging 0.46, while expected heterozygosity (*He*) varied from 0.31 (TH) to 0.58 (LJ), with a mean of 0.43. The number of private alleles (*NPA*) ranged from 0 (most populations) to 5 (LJ), with populations such as LJ and SZ harboring relatively more private alleles. The fixation index (*F*) ranged from −0.33 (NC) to 0.34 (JA, QY), with an overall mean close to zero (−0.01). This suggests that heterozygosity at the species level does not deviate markedly from expectation; however, positive *F* values in populations such as JA and QY may indicate possible inbreeding or bottleneck effects. The LJ population displayed the highest level of genetic diversity (*Na* = 3.92, *He* = 0.58, *I* = 1.03). In contrast, populations such as TH (*Na* = 2.15, *He* = 0.31, *I* = 0.50) and HN (*Na* = 2.15, *He* = 0.32, *I* = 0.51) exhibited relatively low genetic diversity. Populations with small sample sizes, such as HG and HX, also showed low values across all diversity parameters.

### 2.4. Analysis of Molecular Variance in E. senticosus Populations

An analysis of molecular variance (AMOVA) was conducted to examine the genetic variation among different *E. senticosus* populations (Table 4). The results revealed that the majority of total genetic variance (66.3%) resided within populations. Variation among populations contributed 20.3% of the variance, while variance among individuals within populations accounted for the remaining 13.4%. Key population genetic differentiation parameters showed a highly significant genetic differentiation coefficient (*Fst*) of 0.21 (*p* < 0.001) among populations, indicating a moderate level of genetic differentiation across geographic origins. Simultaneously, the inbreeding coefficient within subpopulations (*Fis*) was 0.17 (*p* < 0.001), suggesting a degree of heterozygote deficiency (inbreeding) within populations. In summary, while genetic variation in *E. senticosus* is predominantly distributed within populations, significant genetic differentiation has developed among them, accompanied by signs of inbreeding within populations.

### 2.5. Analysis of Genetic Distance, Genetic Identity, and Genetic Differentiation Among E. senticosus Populations

To further elucidate the genetic relationships and degree of differentiation among different geographical populations of *E. senticosus*, Nei’s genetic distance, genetic identity, and pairwise genetic differentiation coefficients were calculated based on allele frequencies. Analyses of genetic distance and identity (Table 5) revealed considerable variation among the 22 populations. Nei’s genetic distance ranged from 0.025 (RH and HL) to 0.627 (TH and QA), with a mean of 0.282. Genetic identity ranged from 0.528 (QA and HX) to 0.975 (RH and HL), averaging 0.741. In general, geographically proximate populations exhibited smaller genetic distances and higher genetic identities. For instance, populations such as RH, JX, and HL showed mutual genetic distances below 0.05 and genetic identities above 0.95, indicating very close genetic relationships. In contrast, larger genetic distances were observed between some geographically distant or ecologically distinct populations, such as TH, QA, DN, and NC.

The strength of genetic differentiation and gene flow further clarified the patterns of genetic isolation and exchange among populations (Table 6). Pairwise *Fst* values among the 22 populations ranged from 0.015 (RH and HL) to 0.306 (TH and QA), with an average of 0.147. According to Wright’s fixation index [36], approximately 34.8% of population pairs (e.g., SYS–JX, JX–HL, QTH–CH) showed *Fst* < 0.05, indicating negligible differentiation and frequent gene flow. About 30.5% of pairs exhibited moderate differentiation (0.05 < *Fst* < 0.15), while the remaining 34.7% (e.g., TH–QA, JA–HX, HN–SYS) showed *Fst* > 0.15, reflecting high or very high differentiation and restricted genetic exchange. Correspondingly, estimated gene flow (*Nm*) varied widely, from 0.557 (TH and XQ) to 16.270 (RH and HL), with a mean of 2.014. Overall, *Nm* was negatively correlated with *Fst*: population pairs with negligible differentiation (e.g., JX and HL, *Fst* = 0.019) showed very high gene flow (*Nm* = 12.613), indicating active genetic exchange. In contrast, pairs with high differentiation (e.g., TH and QA, *Fst* = 0.306) exhibited low gene flow (*Nm* = 0.568), suggesting barriers to gene flow likely due to geographic or ecological isolation.

In summary, genetic differentiation among geographical populations of *E. senticosus* ranges from negligible to strong, with a pattern broadly correlated with geographic distance. Populations within the core distribution area maintain frequent gene flow and high genetic homogeneity, whereas some peripheral or isolated populations display significant genetic distinctiveness. These findings provide a molecular basis for developing differentiated germplasm collection and targeted conservation strategies, such as prioritizing in situ protection for highly differentiated populations and promoting gene flow among core populations.

### 2.6. Principal Coordinate Analysis and Phylogenetic Analysis of E. senticosus Populations

**Figure 3:**
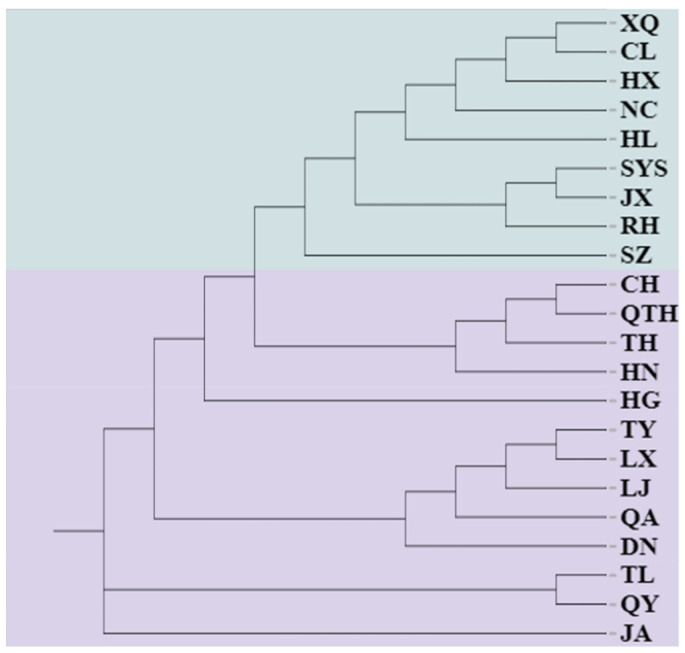
Cluster diagram of 22 *E. senticosus* populations. Different colors represent different clusters.

**Figure 4:**
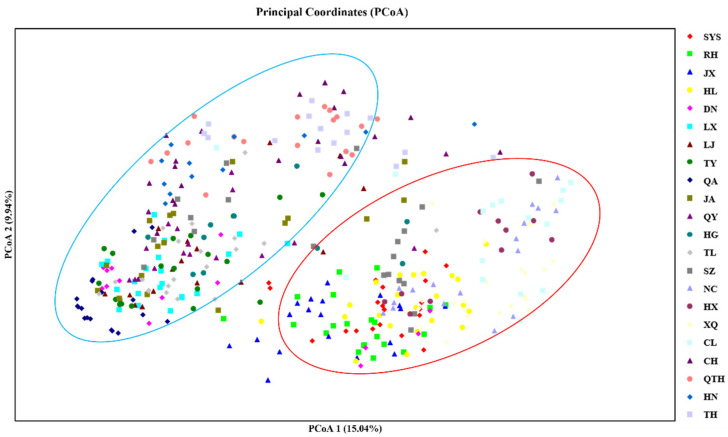
PCoA based on the genetic distance of 405 individuals for *E. senticosus*.

To further clarify the genetic relationships among the natural populations of *E. senticosus*, a neighbor-joining tree constructed from Nei’s genetic distances also clustered the 405 individuals from the 22 populations into two major clades (Figure 3). Group 1 comprised nine populations: XQ, CL, HX, NC, HL, SYS, RH, JX, and SZ. Group 2 consisted of the remaining 13 populations: JA, QY, TL, DN, QA, LJ, CH, TY, HG, HN, TH, QTH, and LX. And a principal coordinate analysis (PCoA) was also performed based on the genetic distances of 405 individuals from the 22 populations (Figure 4). The first and second coordinates explained 15.09% and 9.94% of the total genetic variation, respectively, together accounting for 25.03%. The 22 natural populations were also divided into two major groups, consistent with the grouping pattern revealed by the phylogenetic analysis.

### 2.7. Genetic Structure Analysis of Different Populations of E. senticosus

**Figure 5:**
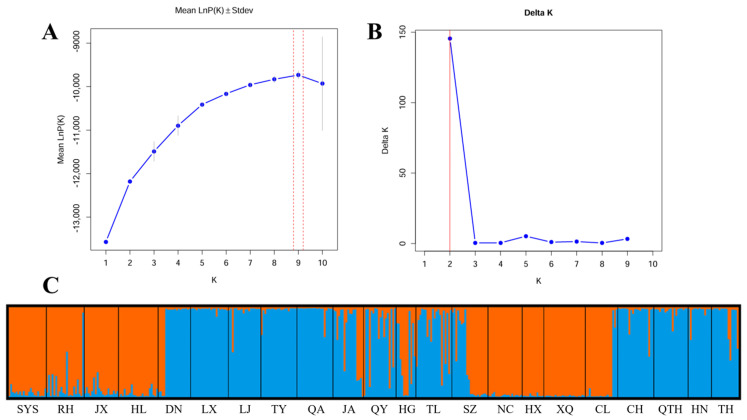
STRUCTURE analysis of the natural population of *E. senticosus* based on 13 EST-SSRs. (**A**) Mean LnP (K). The dotted lines in represent standard deviation (Stdev). (**B**) Delta K. (**C**) Genetic structure map of 22 natural populations of *E. senticosus* based on STRUCTURE analysis (K = 2). Different colors represent different genetic clusters (orange: Cluster I; blue: Cluster II).

The genetic structure of *E. senticosus* populations in Northeast China was analyzed using STRUCTURE [37]. The optimal number of clusters (K) was determined by comparing the rate of change in LnP(K) (Figure 5A) and the ΔK value (Figure 5B). ΔK reached its maximum when K = 2, identifying this as the most likely number of ancestral genetic groups. Based on K = 2, the genetic structure distribution of 405 individuals from the 22 *populations* is displayed in the bar plot (Figure 5C). The individuals were assigned to two genetic clusters, with each population separated by a vertical line and each color representing a distinct genetic cluster. The orange cluster included nine provenance populations: SYS, RH, JX, HL, SZ, NC, HX, XQ, and CL. The blue cluster comprised the remaining 13 provenance populations: DN, LX, LJ, TY, QA, JA, QY, HG, TL, CH, QTH, HN, and TH. This grouping pattern is consistent with the results from the preceding PCoA and phylogenetic tree analyses, which further validates the accuracy of the STRUCTURE analysis.

### 2.8. Isolation by Distance Analysis

**Figure 6:**
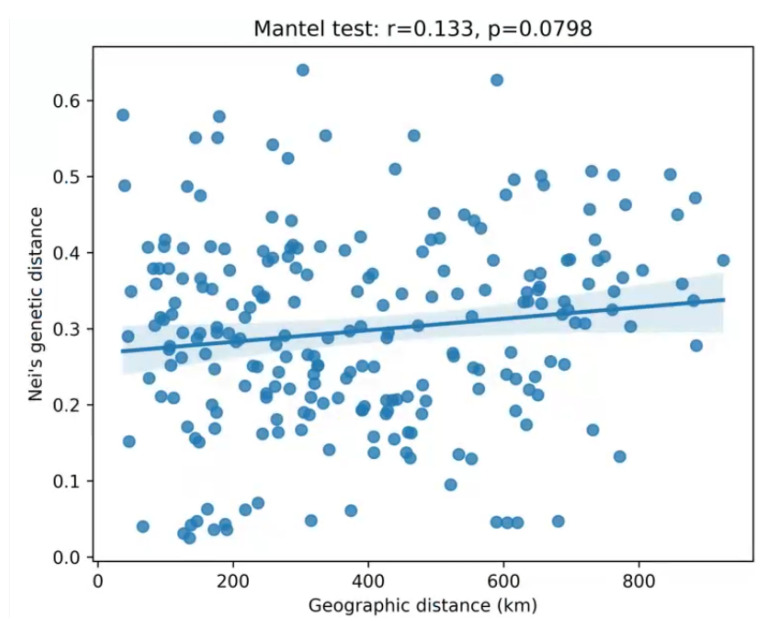
Scatter plot of Mantel test for correlation between genetic distance and geographic distance. The regression line shows a weak positive slope (*r* = 0.133, *p* = 0.0798).

A Mantel test was conducted to evaluate the correlation between genetic distance (Nei’s distance) and geographic distance among the 22 populations. The result revealed a weak positive correlation (*r* = 0.133) that was not statistically significant (*p* = 0.0798, based on 9999 permutations; Figure 6). The scatter plot shows a slightly positive regression slope, but the overall pattern indicates that geographic distance alone does not explain the observed genetic differentiation among populations. The weak IBD signal suggests that geographic distance is not the primary force structuring *E. senticosus* populations.

## 3. Discussion

The development of EST-SSR markers from transcriptomes and their application to population genetics have become an important approach for evaluating the germplasm resources of medicinal plants [38,39]. Compared with genomic SSRs, EST-SSRs are derived from expressed gene regions, which are relatively cost-effective to develop and often exhibit transferability across related species [23]. Moreover, their polymorphism may be potentially linked to functional traits [40]. In this study, we developed and validated a set of polymorphic EST-SSR markers based on full-length transcriptome data of *E. senticosus* for the first time, and conducted a comprehensive analysis of the genetic diversity, population structure, and differentiation patterns of 22 natural germplasm resources from Northeast China. Compared with previously used dominant markers such as AFLP [41], ISSR [42], and RAPD [43] in *E. senticosus*, EST-SSR markers offer advantages including codominant inheritance, good reproducibility, and high cross-laboratory comparability, enabling more accurate estimation of population genetic parameters. This not only provides a reliable tool for the present study but also establishes a molecular foundation for future research on core collection construction, cultivar identification, and association analysis of important traits in *E. senticosus* breeding.

A total of 93,526 EST-SSR loci were identified from the transcriptome of *E. senticosus*. Their abundance was higher than that reported for some tree species such as *J. mandshurica* [24] and *P. koraiensis* [44], suggesting a rich reservoir of microsatellite sequences in the *E. senticosus* genome. Through systematic analysis of the SSR loci, we found that mononucleotide repeats were the most abundant type (27.89%), consistent with observations in many other plants such as *Olea europaea* [23] and *Zanthoxylum bungeanum* [45], highlighting the general enrichment of such simple repeats in plant genomes. Notably, composite SSRs (c- and c*-types, collectively 31.49%) and di-/trinucleotide repeats (collectively 37.27%) also represented substantial proportions, together constituting the majority (>90%) of SSR loci in *E. senticosus*. Studies have shown that trinucleotide repeats often exhibit higher polymorphism and more stable amplification [46]. In this study, we primarily selected 13 trinucleotide-repeat EST-SSR primer pairs, with an average *PIC* of 0.52, confirming the high informativeness of the developed markers for revealing intraspecific genetic variation in *E. senticosus*. Although EST-SSR markers may exhibit slightly lower polymorphism compared to genomic SSRs, the 13 markers developed in this study successfully captured the genetic diversity and structure of *E. senticosus* populations. Future studies could build upon this foundation by developing genome-wide SNP markers through resequencing to enable finer-scale genetic analyses and association mapping. With respect to marker validation, the 13 markers were rigorously tested through multi-round screening across diverse DNA samples, though formal replicate experiments across different experimental batches or by different operators were not performed. Future studies could include such replication to further confirm marker reproducibility and facilitate cross-laboratory comparisons.

Assessment of genetic diversity across 405 samples from 22 geographic populations in Northeast China revealed that *E. senticosus* maintains moderate genetic diversity at the species level (mean *He* = 0.43, *I* = 0.73). This level is lower than that of widespread woody species such as *J. mandshurica* [24] and *Eucalyptus cloeziana* [26], but higher than that of narrowly distributed endangered medicinal plants such as *Camellia fascicularis* [47] and *Paeonia jishanensis* [48]. This suggests that despite rapid population decline due to over-harvesting and habitat destruction, extant populations of *E. senticosus* still retain notable evolutionary potential. However, genetic diversity was highly unevenly distributed among the 22 geographic populations. It is important to note that sample sizes differed among populations (ranging from 8 to 23 individuals, Table 7). Smaller populations (e.g., HG, *n* = 11; HX, *n* = 8) may have slightly underestimated rare allele frequencies and expected heterozygosity. However, the consistent assignment of these small populations to the two major genetic clusters in STRUCTURE analysis (Figure 5C) and their coherent placement in PCoA (Figure 4) indicate that the core findings regarding population structure are robust. Moreover, key comparisons—such as high gene flow among core populations (RH, JX, HL; all *n* > 18) and strong differentiation of peripheral populations (TH, QA; *n* = 15–20)—are based on adequately sampled populations. The LJ population showed the highest values for number of alleles (*Na* = 3.92), expected heterozygosity (*He* = 0.58), and Shannon’s index (*I* = 1.03), and also harbored the most private alleles (*NPA* = 5), indicating high conservation value. In contrast, populations such as TH, HN, and those with small sample sizes like HX and HG exhibited significantly lower genetic diversity. Such disparities likely result from strong anthropogenic disturbance, geographic isolation, and genetic drift leading to a narrow genetic base. Additionally, populations such as JA and QY showed significantly positive fixation indices (*F* = 0.34) and very low observed heterozygosity, indicating inbreeding within these populations. This is likely a direct consequence of severe population size reduction and isolation due to prolonged over-harvesting and habitat fragmentation in these regions, which increases the probability of mating among related individuals and accelerates genetic drift. Conversely, populations such as SYS, RH, and JX exhibited negative *F* values, suggesting heterozygote excess, which could result from heterosis or recent population admixture (e.g., due to the development of under-forest economies introducing individuals from different genetic backgrounds) [49].

Analysis of molecular variance revealed that the majority of genetic variation in *E. senticosus* resides within populations (66.3%), and differentiation among populations was also significant (*Fst* = 0.21), indicating a pattern of high within-population variation coupled with moderate among-population differentiation. A more detailed analysis of pairwise population *Fst* and *Nm* identified areas—most notably centered around geographical regions such as RH, JX, HL, LX, and TY—where *Fst* values were generally below 0.05, while *Nm* values were very high. This suggests extensive gene exchange among populations in these regions, forming a genetically continuous “core distribution area”. In contrast, peripheral or isolated populations such as TH, QA, and NC exhibited high genetic differentiation from the core area and other populations (*Fst* > 0.15, e.g., 0.306 between TH and QA) and very low gene flow (*Nm* ≈ 1 or lower). This pattern likely results from a combination of geographical barriers (e.g., mountain ranges such as the Lesser Khingan Mountains and Changbai Mountains), dispersal limitation beyond effective distances, and possible local environmental adaptation. Notably, the Dongning (DN) population, while not geographically peripheral, also showed relatively high genetic distances and *Fst* values with many other populations, suggesting a distinct evolutionary history or strong local climatic and environmental adaptation.

The weak and non-significant isolation-by-distance pattern detected by the Mantel test (*r* = 0.133, *p* = 0.080) further supports the notion that geographic distance is not the primary driver of genetic differentiation in *E. senticosus*. This finding aligns with the observation that populations within the core distribution area (e.g., RH, JX, HL) exhibit extremely high gene flow (*Nm* > 10) despite spanning hundreds of kilometers, indicating that gene exchange is not strongly limited by geographic proximity. Conversely, the pronounced genetic divergence of peripheral populations (e.g., TH, QA, DN) is unlikely to result from continuous distance-limited dispersal, given the weak IBD signal. Instead, it more likely reflects genetic drift in small, isolated populations at the edge of the species’ range, combined with limited gene flow due to landscape barriers (e.g., the Changbai Mountains). This interpretation is consistent with the reduced genetic diversity (*He* = 0.31–0.36) and positive fixation indices observed in these populations (Table 3).

Results from PCoA, neighbor-joining clustering, and STRUCTURE analyses were highly concordant, consistently dividing the 22 populations into two major genetic clusters. Cluster I primarily includes populations from central-eastern Heilongjiang Province (e.g., SYS, SZ, RH, JX), whereas Cluster II mainly comprises populations from eastern Jilin Province and southern Heilongjiang (e.g., JA, TH, TL, DN). *E. senticosus* is primarily distributed across Northeast China, the Russian Far East, and the Korean Peninsula. The clear divergence into two major genetic clusters observed across all sampled populations represents the primary genetic structure of *E. senticosus* in Northeast China, with populations clearly partitioned into two geographically distinct groups. While direct fossil or paleoclimatic evidence specific to *E. senticosus* is lacking, it is plausible that the two genetic lineages originated from distinct glacial refugia [50], potentially located in the Changbai Mountains and the Lesser Khingan Mountains or Sikhote-Alin region, followed by postglacial range expansion and secondary contact. However, we acknowledge that this interpretation remains hypothetical and requires further testing using approaches such as paleodistribution modeling or population genomic analyses with fossil-calibrated phylogenies.

As a traditionally important medicinal and edible plant in Northeast China, increasing market demand has led to uncontrolled harvesting of wild *E. senticosus*, contributing to its endangered status in the wild. Based on our findings, we propose the following science-based strategies for its conservation, utilization, and management: (1) Conservation priorities: Populations with high genetic diversity (e.g., LJ) should be priority sites for in situ conservation. Genetically distinct populations like TH, QA, and particularly DN—which shows unique genetic ancestry despite its central location, possibly due to local adaptation or a unique historical colonization path—should be managed as independent conservation units. For populations exhibiting inbreeding (e.g., JA), ex situ conservation collections should be established with founders sourced from a genetically diverse subset of individuals within the same genetic cluster (Cluster II) to maintain adaptive integrity. Additionally, habitat restoration to reduce fragmentation could facilitate natural gene flow. (2) Germplasm collection strategy: Germplasm repositories should systematically preserve representative samples from both major genetic clusters. Collection efforts should be intensified in peripheral and highly differentiated regions to capture rare genetic variants. (3) Breeding parent selection: Crossing superior individuals with large genetic distances (e.g., from different clusters) should be prioritized to maximize heterosis potential. For example, parents could be selected from Cluster I (e.g., JX) and Cluster II (e.g., JA). Furthermore, conservation planning must consider the ongoing challenge of climate change. The identified genetic lineages and adaptive potential stored within diverse populations, particularly those at climatic margins (e.g., high-elevation TH population), will be crucial for the species’ resilience. In situ conservation networks should aim to protect populations across environmental gradients to preserve adaptive genetic variation. Future conservation strategies may need to incorporate assisted migration—the facilitated movement of germplasm from genetically distinct but climatically analogous populations. This approach, guided by the genetic structure revealed in this study, could enhance adaptive capacity in vulnerable populations. In summary, this study provides a systematic assessment of the genetic background of *E. senticosus* germplasm and identifies genetic diversity hotspots, endangered populations, and evolutionarily distinct lineages.

## 4. Materials and Methods

### 4.1. Acquisition of E. senticosus Materials and DNA Extraction

This study conducted an extensive collection and preservation of *E. senticosus* germplasm resources across Northeast China, obtaining a total of 405 representative samples from 22 populations (Table 7). Within each population, sampling sites were spaced at least 100 m apart. Mature leaves were collected, temporarily stored on ice, and transported to the laboratory where they were subsequently kept at −40 °C until DNA extraction. Sampling aimed to cover the major distribution range and ecological gradients of *E. senticosus* in Northeast China. The target sample size was ≥15 individuals per population where possible. Variations in final sample size (Table 7) resulted from natural population density differences and accessibility constraints in remote areas. The potential influence of smaller sample sizes on diversity estimates is acknowledged in the discussion. Genomic DNA was isolated from the leaf tissue using a modified cetyltrimethylammonium bromide (CTAB) rapid extraction protocol: (1) Prepare CTAB extraction buffer (containing 5% β-mercaptoethanol and 1% PVP). (2) Transfer 0.2 g of finely ground plant tissue into a new 1.5 mL centrifuge tube, quickly add 800 μL of prepared CTAB buffer, and vortex vigorously for 1 min. (3) Incubate the tube in a 65 °C water bath for 15 min, inverting gently every 5 min. After incubation, centrifuge at 12,500 rpm for 5 min. (4) Transfer 700 μL of the supernatant to a new 1.5 mL centrifuge tube, add 700 μL of chloroform, vortex for 30 s, and centrifuge at 12,500 rpm for 5 min. (5) Transfer 600 μL of the aqueous upper phase to a new tube, add another 600 μL of chloroform, vortex for 30 s, and centrifuge at 12,500 rpm for 5 min. (6) Transfer 400 μL of the upper aqueous phase into a fresh tube, add 800 μL of absolute ethanol (2× volume), mix gently by inverting for 1 min, and centrifuge at 13,500 rpm for 10 min. (7) Wash the pellet twice with 600 μL of 70% ethanol solution each time. (8) Discard the supernatant and retain the pellet. Air-dry the pellet in a fume hood until it appears translucent. (9) Dissolve the pellet in 50 μL of deionized water. Label clearly, measure DNA concentration, and store at −20 °C. The quality of the extracted DNA was assessed via 1% agarose gel electrophoresis (110 V), visualized using a gel imaging system (Tanon, Shanghai, China; 2500R). DNA concentration and purity were measured with a micro-spectrophotometer (KAIAO, Beijing, China; K5600).

### 4.2. Development of EST-SSR Primers for E. senticosus

The raw full-length transcriptome sequencing data used for developing EST-SSR markers for *E. senticosus* have been deposited in the Genome Sequence Archive (GSA) database of the China National Center for Bioinformation (CNCB) under BioProject accession number PRJCA055497. SSR loci were identified from *E. senticosus* unigene sequences using the MicroSatellite identification tool (MISA) [51]. To balance locus abundance and polymorphism potential, the following search criteria were applied: a minimum of 10 repeats for mononucleotide motifs, six repeats for dinucleotide motifs, and five repeats for tri-, tetra-, penta-, and hexanucleotide motifs. SSR loci with sufficient flanking sequence length and quality were selected for primer design using Primer 3 software [52]. Design parameters were set as follows: primer length 18–24 bp; GC content 40–60%; annealing temperature (Tm) 55–60 °C; expected PCR product size 100–350 bp.

After initial design, primers were further screened through online tools and manual inspection. First, a preliminary filter was applied in Excel to: (1) remove primers with missing information; (2) retain SSR primers targeting di-, tri-, penta-, and hexanucleotide repeats; (3) exclude compound repeat sequences; and (4) select products longer than 150 bp to facilitate clear visualization during electrophoretic separation. Following Excel-based screening, primer properties—including annealing temperature, GC content, and PCR suitability—were calculated using the online PCR Primer Stats tool (https://www.detaibio.com/sms2/pcr_primer_stats.html, accessed on 6 March 2026). Redundant and low-quality primer pairs were removed. Finally, 96 candidate primer pairs covering different repeat types were randomly chosen for synthesis. A universal M13 sequence (5′-TGTAAAACGACGGCCAGT-3′) labeled with fluorescent dyes (FAM, TAMRA, ROX, HEX) was appended to the 5′ end of each forward primer. All primers were commercially synthesized by Heilongjiang Jiansu Gene Technology Co., Ltd. (Harbin, China).

### 4.3. Primer Screening and PCR Amplification

For primer screening via PCR, amplification was performed using BGI 2× Super PCR Mix (with dye, green). Each 20 μL reaction contained 10 μL PCR Mix, 0.8 μL forward primer, 0.8 μL reverse primer, 2 μL template DNA, and 6.4 μL ddH_2_O. The thermal cycling profile consisted of initial denaturation at 94 °C for 5 min; 35 cycles of 94 °C for 30 s, 60 °C for 30 s, and 72 °C for 20 s; followed by a final extension at 72 °C for 10 min. Screening was conducted in multiple successive rounds. First, two high-quality DNA samples were used for conventional PCR, and primers that failed to produce clear bands were eliminated. Subsequently, four additional DNA samples from different populations were tested, and non-amplifying primers were again discarded. This iterative process was repeated with eight further DNA samples from distinct sources until all remaining primers consistently amplified bands across all tested samples.

Subsequently, primers labeled with different fluorescent dyes were used for SSR amplification. PCR products were wrapped in foil to protect from light and sent to Sangon Biotech (Shanghai) Co., Ltd. (Shanghai, China) for capillary electrophoresis-based polymorphism detection. A final set of 13 polymorphic EST-SSR primer pairs was selected (Table 8). These 13 primer pairs were then used to amplify all 405 individuals from the 22 natural populations of *E. senticosus*. The resulting amplification products were analyzed by capillary electrophoresis, and the resulting data were used to assess the population genetic characteristics of *E. senticosus*.

### 4.4. Data Analysis

Raw capillary electrophoresis peak data were processed for allele calling using GeneMarker software (version 1.65) [53]. The genotyping results were converted into GenePOP format using MS Tools for subsequent population genetic analyses [24]. Genetic diversity parameters—including the observed number of alleles (*Na*), effective number of alleles (*Ne*), observed heterozygosity (*Ho*), expected heterozygosity (*He*), and Shannon’s information index (*I*)—were calculated for each locus and population using GenAIEx version 6.502 [54]. The same software was used to perform analysis of molecular variance (AMOVA) and principal coordinate analysis (PCoA). Additionally, fixation index (*F*), inbreeding coefficient within subpopulations (*Fis*), genetic differentiation among populations (*Fst*), and gene flow (Nm) were estimated at the provenance level. Polymorphism information content (*PIC*) for each EST-SSR locus was calculated using PICcalc software (version 0.6) [55]. To investigate the population genetic structure of *E. senticosus*, data were first converted into the STRUCTURE input format using CONVERT version 1.31 [56]. A Bayesian clustering analysis was then implemented in STRUCTURE version 2.3.4 [37], using the admixture model with correlated allele frequencies. The parameter λ (the Dirichlet parameter for allele frequencies) was set to 1.0. For each K (ranging from 1 to 10), three independent runs were performed, each consisting of a burn-in period of 50,000 iterations followed by 100,000 Markov chain Monte Carlo (MCMC) iterations for data collection. The Structure Selector online platform (https://lmme.ac.cn/StructureSelector/index.html, accessed on 6 March 2026) was used to process the results, and the optimal K was determined based on the ΔK method [57]. A neighbor-joining phylogenetic tree was constructed using PowerMarker version 3.25 [58] based on Nei’s genetic distance. The resulting tree was visualized and annotated using MEGA version 7.0 [59] and the Interactive Tree of Life (iTOL) online tool (https://itol.embl.de, accessed on 6 March 2026) [60]. To test for isolation by distance (IBD), a Mantel test was performed using the R package vegan (version 2.6-4). Pairwise Nei’s genetic distances (Table 5, below diagonal) were extracted and converted into a distance matrix. Geographic distances (Euclidean distance, km) were calculated from the latitude and longitude coordinates of each population (Table 7) using the geosphere package or via the Haversine formula. The Mantel test was conducted with 9999 permutations, using Pearson’s correlation coefficient as the test statistic. The correlation coefficient (*r*) and its significance (*p*-value) were computed to assess the relationship between genetic and geographic distance.

## 5. Conclusions

Based on transcriptome sequencing data of *E. senticosus*, this study successfully developed 13 highly polymorphic (mean *PIC* = 0.52) EST-SSR markers. Using these markers, we systematically evaluated the genetic diversity and population genetic structure of 405 germplasm samples collected from 22 geographical populations across Northeast China. The results showed that *E. senticosus* maintains moderate genetic diversity at the species level, but significant variation exists among different geographical populations. Analysis of molecular variance revealed that the majority of genetic variation resides within populations (66.3%), while moderate genetic differentiation also exists among populations (*Fst* = 0.21). Gene flow analysis indicated frequent genetic exchange and high genetic homogeneity among populations within the core distribution area (e.g., the region encompassing RH, JX, and HL). Through PCoA, neighbor-joining clustering, and genetic structure analysis, the 22 populations were classified into two major genetic clusters, potentially corresponding to different historical refugia. Notably, the DN population exhibited a distinct genetic signature, warranting special conservation attention. This study also identified populations with high inbreeding risk (e.g., JA) and those with extremely high genetic connectivity (e.g., RH-HL). By employing EST-SSR marker analysis, this study clarifies the genetic background, variation patterns, and population relationships of the natural germplasm resources of *E. senticosus*. The findings provide a critical basis for the scientific conservation, efficient collection, and preservation of its germplasm resources, and also establish a solid genetic foundation for subsequent cross-breeding and selection of superior germplasm. Future research should build upon this foundation by: (1) conducting functional gene association studies using these EST-SSRs or linked SNPs; (2) performing whole-genome resequencing of representative individuals from both lineages to unravel demographic history and identify selection signatures; and (3) integrating ecological niche modeling with genetic data to predict climate change impacts and design dynamic conservation strategies.

## Figures and Tables

**Table 1:** Number of repeats of different sequence units of EST-SSR loci from *E. senticosus*.

Repeats	Mononucleotide	Dinucleotide	Trinucleotide	Tetranucleotide	Pentanucleotide	Hexanucleotides	Composite	Total	Percentage
5			10,942	1173	337	498		12,950	13.85
6		8088	4378	335	107	247		13,155	14.07
7		4051	1838	183	12	57		6141	6.57
8		2787	866	62		31		3746	4.01
9		1530	374	35		24		1963	2.10
10	17,989			9	1	3		18,002	19.25
11	8094			11				8105	8.67
12				2		4		6	0.006
13				1				1	0.001
14				1	3	1		5	0.005
Others				1			29,451	29,452	31.49
Total	26,083	16,456	18,398	1813	460	865	29,451	93,526	100

**Table 2:** Genetic diversity parameters of 13 polymorphic EST-SSR loci in *E. senticosus* populations.

Primer	*Na*	*Ne*	*I*	*Ht*	*Ho*	*He*	*uHe*	*F*	*PIC*	*Fis*	*Fit*	*Fst*	*Nm*
Ese-6	6	1.71	0.60	0.43	0.52	0.39	0.40	−0.29	0.37	−0.35	−0.20	0.11	1.95
Ese-7	8	2.67	1.02	0.72	0.51	0.55	0.57	0.11	0.68	0.09	0.30	0.23	0.84
Ese-9	8	2.08	0.86	0.55	0.41	0.48	0.49	0.19	0.52	0.15	0.26	0.13	1.66
Ese-14	4	1.60	0.44	0.40	0.53	0.31	0.32	−0.60	0.32	−0.70	−0.33	0.22	0.90
Ese-15	5	1.59	0.57	0.56	0.26	0.32	0.33	0.29	0.49	0.19	0.54	0.43	0.32
Ese-20	3	1.67	0.57	0.41	0.27	0.38	0.39	0.33	0.33	0.30	0.35	0.07	3.16
Ese-22	13	3.01	1.25	0.80	0.95	0.66	0.68	−0.45	0.77	−0.44	−0.19	0.17	1.19
Ese-24	4	1.81	0.65	0.65	0.11	0.43	0.45	0.73	0.57	0.74	0.82	0.33	0.50
Ese-32	4	1.97	0.73	0.53	0.37	0.46	0.48	0.19	0.43	0.21	0.31	0.13	1.69
Ese-55	7	2.10	0.77	0.74	0.93	0.51	0.54	−0.83	0.67	−0.81	−0.26	0.31	0.57
Ese-65	8	1.78	0.64	0.71	0.24	0.35	0.37	0.36	0.64	0.31	0.66	0.51	0.24
Ese-72	8	2.48	0.96	0.77	0.58	0.55	0.57	−0.11	0.74	−0.06	0.25	0.29	0.61
Ese-74	8	1.44	0.40	0.29	0.33	0.24	0.25	−0.35	0.27	−0.39	−0.14	0.18	1.14
Mean	6.62	1.99	0.73	0.58	0.46	0.43	0.45	−0.03	0.52	−0.06	0.18	0.24	1.14

Note: *Na*, Total number of alleles; *Ne*, Effective number of alleles; *I*, Shannon’s Information Index; *Ht*, Total gene diversity; *Ho*, Observed heterozygosity; *He*, Expected heterozygosity; *uHe*, Unbiased expected heterozygosity; *F*, Fixation index; *PIC*, Polymorphism information content; *Fis*, Inbreeding coefficient within subpopulations; *Fit*, Total inbreeding coefficient; *Fst*, Genetic differentiation coefficient; *Nm*, Gene flow.

**Table 3:** Genetic diversity parameters of different populations of *E. senticosus*.

Provenance	*N*	*Na*	*Ne*	*I*	*Ho*	*He*	*uHe*	*F*	*NPA*
Shuangyashan (SYS)	18.85	3	2.08	0.82	0.62	0.49	0.5	−0.27	1
Raohe (RH)	19.23	3	2.15	0.84	0.62	0.51	0.52	−0.23	1
Jixi (JX)	18.08	3.38	2.31	0.87	0.63	0.51	0.52	−0.24	2
Hulin (HL)	17.92	2.85	2.13	0.79	0.62	0.49	0.5	−0.29	0
Dongning (DN)	17.31	2.69	2.06	0.74	0.55	0.45	0.46	−0.13	0
Langxiang (LX)	20.85	3	2.18	0.83	0.56	0.5	0.51	−0.06	0
Linjiang (LJ)	16.85	3.92	2.52	1.03	0.5	0.58	0.6	0.12	5
Tangyuan (TY)	19	3.31	2.33	0.91	0.56	0.53	0.54	0	0
Qingan (QA)	19.69	2.85	1.93	0.63	0.47	0.36	0.36	−0.14	0
Jian (JA)	14.77	2.77	1.82	0.65	0.29	0.38	0.39	0.34	0
Qingyuan (QY)	16.38	3.23	2.28	0.86	0.37	0.49	0.51	0.34	2
Hegang (HG)	9.54	2.31	1.63	0.55	0.43	0.35	0.37	0	0
Tieli (TL)	19.15	3.31	2.03	0.79	0.41	0.45	0.46	0.16	2
Shangzhi (SZ)	17.15	3.08	2.28	0.86	0.42	0.51	0.52	0.14	4
Nancha (NC)	14	2.38	1.82	0.62	0.52	0.39	0.4	−0.33	0
Hongxing (HX)	8.92	2.62	1.76	0.68	0.47	0.41	0.43	−0.15	0
Xinqing (XQ)	16.92	2.54	1.65	0.59	0.41	0.36	0.37	−0.09	0
Cuiluan (CL)	11.77	2.77	1.89	0.68	0.41	0.41	0.43	−0.02	0
Chaihe (CH)	15.92	2.46	1.96	0.64	0.36	0.39	0.41	0.05	0
Qitaihe (QTH)	14.92	2.69	1.78	0.59	0.3	0.35	0.37	0.21	0
Huinan (HN)	11.38	2.15	1.66	0.51	0.36	0.32	0.34	−0.04	0
Tonghua (TH)	11.77	2.15	1.57	0.5	0.25	0.31	0.33	0.29	1
Mean	15.93	2.84	1.99	0.73	0.46	0.43	0.45	−0.01	0.82

Note: *N*, Mean number of sampled individuals per locus (adjusted for missing data); *Na*, Mean number of alleles; *Ne*, Effective number of alleles; *I*, Shannon’s Information Index; *Ho*, Observed heterozygosity; *He*, Expected heterozygosity; *uHe*, Unbiased expected heterozygosity; *F*, Fixation index; *NPA*, Number of private alleles.

**Table 4:** AMOVA for the *E. senticosus* populations.

Source	df	SS	MS	VC	PV/%	*Fst*	*Fis*
Among Pops	21	738.09	35.15	0.85	20.3		
Among Individual	383	1492.68	3.90	0.56	13.4		
Within Individual	405	1124.00	2.78	2.78	66.3		
Total	809	3354.76		4.19	100	0.21 ***	0.17 ***

Note: *Fst*, genetic differentiation coefficient; *Fis*, inbreeding coefficient; *** denotes *p* < 0.001.

**Table 5:** Nei’s genetic distance (below diagonal) and genetic identity (above diagonal) among different populations of *E. senticosus*.

	SYS	RH	JX	HL	DN	LX	LJ	TY	QA	JA	QY	HG	TL	SZ	NC	HX	XQ	CL	CH	QTH	HN	TH
**SYS**	*	0.94	0.965	0.965	0.715	0.744	0.714	0.727	0.592	0.698	0.735	0.716	0.73	0.787	0.856	0.827	0.835	0.849	0.711	0.73	0.621	0.677
**RH**	0.062	*	0.953	0.975	0.743	0.778	0.739	0.778	0.636	0.714	0.757	0.684	0.745	0.768	0.811	0.829	0.813	0.849	0.689	0.678	0.63	0.637
**JX**	0.036	0.048	*	0.958	0.684	0.785	0.738	0.778	0.575	0.649	0.704	0.643	0.69	0.756	0.799	0.798	0.779	0.824	0.659	0.665	0.575	0.637
**HL**	0.036	0.025	0.043	*	0.705	0.749	0.717	0.747	0.6	0.674	0.723	0.674	0.706	0.75	0.83	0.81	0.814	0.856	0.667	0.693	0.606	0.633
**DN**	0.335	0.297	0.379	0.349	*	0.791	0.825	0.817	0.814	0.909	0.879	0.825	0.872	0.796	0.668	0.677	0.686	0.659	0.751	0.717	0.707	0.658
**LX**	0.295	0.251	0.243	0.288	0.235	*	0.955	0.961	0.758	0.773	0.792	0.694	0.748	0.75	0.614	0.64	0.622	0.744	0.806	0.753	0.765	0.715
**LJ**	0.336	0.303	0.304	0.333	0.192	0.046	*	0.956	0.782	0.81	0.818	0.727	0.787	0.738	0.609	0.605	0.602	0.715	0.82	0.779	0.77	0.738
**TY**	0.319	0.251	0.251	0.291	0.202	0.04	0.045	*	0.781	0.776	0.808	0.684	0.777	0.71	0.559	0.581	0.56	0.703	0.779	0.745	0.729	0.701
**QA**	0.524	0.452	0.554	0.51	0.206	0.277	0.246	0.247	*	0.84	0.802	0.72	0.791	0.74	0.577	0.528	0.576	0.614	0.663	0.666	0.67	0.534
**JA**	0.359	0.337	0.432	0.395	0.095	0.257	0.211	0.253	0.174	*	0.959	0.876	0.954	0.849	0.677	0.677	0.686	0.705	0.815	0.789	0.766	0.706
**QY**	0.308	0.278	0.351	0.325	0.129	0.234	0.2	0.213	0.221	0.042	*	0.846	0.956	0.854	0.704	0.698	0.677	0.723	0.829	0.802	0.761	0.732
**HG**	0.334	0.38	0.442	0.395	0.193	0.366	0.319	0.379	0.328	0.132	0.167	*	0.86	0.777	0.699	0.686	0.701	0.665	0.767	0.745	0.706	0.659
**TL**	0.315	0.295	0.371	0.349	0.137	0.29	0.24	0.252	0.235	0.047	0.045	0.151	*	0.799	0.666	0.669	0.666	0.684	0.802	0.811	0.707	0.691
**SZ**	0.24	0.264	0.279	0.288	0.228	0.287	0.303	0.342	0.302	0.163	0.158	0.252	0.225	*	0.851	0.878	0.869	0.846	0.827	0.768	0.811	0.718
**NC**	0.156	0.209	0.224	0.187	0.403	0.488	0.496	0.581	0.551	0.391	0.351	0.359	0.407	0.162	*	0.931	0.954	0.843	0.675	0.667	0.643	0.613
**HX**	0.19	0.188	0.226	0.211	0.39	0.447	0.503	0.542	0.64	0.39	0.359	0.377	0.402	0.13	0.071	*	0.97	0.845	0.711	0.657	0.693	0.624
**XQ**	0.181	0.207	0.25	0.206	0.376	0.475	0.507	0.579	0.551	0.377	0.39	0.355	0.406	0.141	0.047	0.031	*	0.859	0.693	0.665	0.689	0.605
**CL**	0.164	0.164	0.193	0.155	0.417	0.295	0.336	0.352	0.487	0.349	0.325	0.408	0.379	0.167	0.171	0.169	0.152	*	0.784	0.768	0.764	0.736
**CH**	0.341	0.372	0.417	0.406	0.287	0.215	0.198	0.25	0.41	0.205	0.188	0.266	0.221	0.19	0.393	0.342	0.367	0.243	*	0.939	0.941	0.872
**QTH**	0.315	0.389	0.408	0.366	0.332	0.283	0.249	0.294	0.406	0.237	0.22	0.294	0.21	0.263	0.405	0.421	0.408	0.264	0.063	*	0.874	0.825
**HN**	0.476	0.463	0.554	0.501	0.346	0.268	0.262	0.316	0.401	0.267	0.273	0.348	0.346	0.21	0.442	0.367	0.373	0.269	0.061	0.135	*	0.811
**TH**	0.39	0.45	0.45	0.457	0.419	0.335	0.304	0.355	0.627	0.349	0.312	0.417	0.37	0.331	0.489	0.472	0.502	0.307	0.137	0.192	0.209	*

Note: Genetic distance measures the degree of genetic difference between two populations; a larger value indicates greater genetic divergence, while a smaller value indicates very little genetic divergence. Genetic identity measures the degree of genetic similarity between two populations; a larger value indicates higher identity, while a smaller value indicates lower identity. Genetic distance and genetic identity are complementary: a smaller genetic distance corresponds to higher genetic identity, and a larger genetic distance corresponds to lower genetic identity. * indicates diagonal elements (self-comparisons).

**Table 6:** Analysis of genetic differentiation coefficient (below diagonal) and gene flow (above diagonal) among different populations of *E. senticosus*.

	SYS	RH	JX	HL	DN	LX	LJ	TY	QA	JA	QY	HG	TL	SZ	NC	HX	XQ	CL	CH	QTH	HN	TH
**SYS**	*	8.792	11.818	12.239	1.586	2.408	2.342	2.239	0.935	1.278	1.837	1.416	1.667	2.437	1.86	1.791	1.689	2.702	1.364	1.402	0.953	0.935
**RH**	0.028	*	10.374	16.27	1.849	2.932	2.729	3.085	1.078	1.367	2.101	1.227	1.84	2.33	1.565	1.818	1.548	2.751	1.294	1.185	0.986	0.851
**JX**	0.021	0.024	*	12.613	1.375	2.97	2.519	2.882	0.881	1.083	1.648	1.091	1.487	2.106	1.427	1.577	1.326	2.25	1.133	1.117	0.828	0.814
**HL**	0.02	0.015	0.019	*	1.489	2.486	2.299	2.461	0.959	1.149	1.725	1.191	1.514	2.044	1.633	1.612	1.515	2.746	1.121	1.17	0.874	0.787
**DN**	0.136	0.119	0.154	0.144	*	2.16	2.955	2.527	1.986	3.576	3.655	1.992	3.089	2.39	1.024	1.099	1.073	1.185	1.559	1.303	1.194	0.877
**LX**	0.094	0.079	0.078	0.091	0.104	*	11.841	14.803	1.501	1.739	2.459	1.362	1.985	2.197	0.958	1.091	0.966	1.897	1.96	1.593	1.468	1.032
**LJ**	0.096	0.084	0.09	0.098	0.078	0.021	*	12.363	1.771	2.251	3.266	1.634	2.556	2.438	1.081	1.141	1.039	1.818	2.318	1.882	1.683	1.229
**TY**	0.1	0.075	0.08	0.092	0.09	0.017	0.02	*	1.712	1.846	2.87	1.312	2.35	1.981	0.877	0.984	0.855	1.664	1.814	1.572	1.352	1.042
**QA**	0.211	0.188	0.221	0.207	0.112	0.143	0.124	0.127	*	1.929	1.794	1.148	1.713	1.369	0.692	0.638	0.717	0.935	0.972	0.938	0.936	0.568
**JA**	0.164	0.155	0.188	0.179	0.065	0.126	0.1	0.119	0.115	*	8.177	2.761	7.61	2.785	0.832	0.9	0.873	1.215	2.123	1.675	1.481	0.899
**QY**	0.12	0.106	0.132	0.127	0.064	0.092	0.071	0.08	0.122	0.03	*	2.685	10.58	3.503	1.115	1.19	1.027	1.551	2.71	2.132	1.754	1.246
**HG**	0.15	0.169	0.186	0.174	0.112	0.155	0.133	0.16	0.179	0.083	0.085	*	2.756	1.766	0.901	0.884	0.91	1.023	1.414	1.23	1.019	0.744
**TL**	0.13	0.12	0.144	0.142	0.075	0.112	0.089	0.096	0.127	0.032	0.023	0.083	*	2.312	0.933	1.022	0.945	1.269	2.112	2.058	1.246	0.952
**SZ**	0.093	0.097	0.106	0.109	0.095	0.102	0.093	0.112	0.154	0.082	0.067	0.124	0.098	*	1.931	2.468	2.069	3.237	2.483	1.742	2.092	1.153
**NC**	0.119	0.138	0.149	0.133	0.196	0.207	0.188	0.222	0.265	0.231	0.183	0.217	0.211	0.115	*	1.753	1.975	1.513	0.828	0.796	0.708	0.575
**HX**	0.122	0.121	0.137	0.134	0.185	0.186	0.18	0.203	0.281	0.217	0.174	0.221	0.197	0.092	0.125	*	2.139	1.623	1.021	0.83	0.881	0.621
**XQ**	0.129	0.139	0.159	0.142	0.189	0.206	0.194	0.226	0.259	0.223	0.196	0.216	0.209	0.108	0.112	0.105	*	1.62	0.898	0.805	0.82	0.557
**CL**	0.085	0.083	0.1	0.083	0.174	0.116	0.121	0.131	0.211	0.171	0.139	0.196	0.165	0.072	0.142	0.133	0.134	*	1.596	1.477	1.435	0.955
**CH**	0.155	0.162	0.181	0.182	0.138	0.113	0.097	0.121	0.205	0.105	0.084	0.15	0.106	0.091	0.232	0.197	0.218	0.135	*	5.355	5.185	1.791
**QTH**	0.151	0.174	0.183	0.176	0.161	0.136	0.117	0.137	0.21	0.13	0.105	0.169	0.108	0.126	0.239	0.231	0.237	0.145	0.045	*	2.225	1.233
**HN**	0.208	0.202	0.232	0.222	0.173	0.146	0.129	0.156	0.211	0.144	0.125	0.197	0.167	0.107	0.261	0.221	0.234	0.148	0.046	0.101	*	1.165
**TH**	0.211	0.227	0.235	0.241	0.222	0.195	0.169	0.194	0.306	0.218	0.167	0.252	0.208	0.178	0.303	0.287	0.31	0.207	0.123	0.169	0.177	*

Note: The genetic differentiation coefficient (*Fst*) measures the degree of genetic divergence among populations, representing the proportion of total genetic variation attributable to differences between populations. Values of 0–0.05 indicate negligible differentiation, 0.05–0.15 moderate differentiation, 0.15–0.25 high differentiation, and >0.25 very high differentiation. Gene flow (*Nm*) denotes the effective number of migrants per generation; *Nm* > 1 indicates substantial gene flow, while *Nm* < 1 suggests limited gene flow. * indicates diagonal elements (self-comparisons).

**Table 7:** Environmental factors of different *E. senticosus* populations.

No.	Provenance	Latitude (N)	Longitude (E)	Elevation /m	Annual Mean Temperature/°C	Annual Precipitation/mm	Test Number
1	Hongxing (HX)	49°13′44.01″	129°37′03.21″	415	0–1	550–650	19
2	Xinqing (XQ)	48°18′33.72″	128°35′55.13″	475	1–2	600–700	23
3	Cuiluan (CL)	47°59′10.00″	128°12′30.31″	372	1–2	550–650	18
4	Hegang (HG)	47°32′27.54″	130°19′46.13″	226	2–3	550–650	11
5	Tieli (TL)	47°10′54.24″	128°25′6.97″	200	2–3	550–650	20
6	Nancha (NC)	47°06′19.76″	129°23′24.65″	270	1–2	550–650	19
7	Langxiang (LX)	46°57′43.15″	128°54′41.41″	362	0–1	550–600	20
8	Tangyuan (TY)	46°54′23.60″	129°46′55.30″	450	3–4	550–600	20
9	Qingan (QA)	46°53′44.51″	127°30′58.29″	150	2–3	500–550	20
10	Raohe (RH)	46°48′7.24″	134°2′5.44″	200	2–3	600–700	20
11	Shuangyashan (SYS)	46°42′04.25″	131°11′11.35″	406	3–4	550–600	21
12	Qitaihe (QTH)	45°52′24.13″	131°03′12.64″	240	3–4	550–600	12
13	Hulin (HL)	45°48′43.14″	133°0′28.29″	100	2–4	550–600	22
14	Shangzhi (SZ)	45°18′48.70″	127°34′44.86″	500	2–3	600–700	21
15	Jixi (JX)	44°59′52.68″	130°54′1.56″	200	4–5	550–600	19
16	Dongning (DN)	44°05′19.86″	131°14′22.64″	600	5–6	550–600	18
17	Chaihe (CH)	44°47′15.11″	129°40′46.23″	500	3–4	600–650	21
18	Huinan (HN)	42°42′36.74″	126°01′23.16″	400	4–5	700–800	13
19	Qingyuan (QY)	42°09′54.15″	124°58′38.18″	500	6–7	800–900	18
20	Linjiang (LJ)	41°50′41.93″	126°58′01.66″	406	3–4	800–900	18
21	Tonghua (TH)	41°42′13.18″	125°58′23.00″	949	5–6	750–850	15
22	Jian (JA)	41°16′47.85″	126°08′10.54″	610	6–7	900–1000	17

**Table 8:** EST-SSR primers information of *E. senticosus*.

Primer	SSR Type	Primer Sequences (5′-3′)	Size (bp)
Ese-6	(GAT)9	F-5′-TCCCCAAAGCTTGTCTGACC-3′	296
		R-5′-AGCCAACCAAGAAACCAGGT-3′	
Ese-7	(ATA)9	F-5′-CCGTGAAGGGACTGAAAGGA-3′	269
		R-5′-CAGCAGCACCTTCATCAAGC-3′	
Ese-9	(GCT)8	F-5′-TGGCAGACCTGAAAGTGAAAGA-3′	220
		R-5′-CTGCTCCAGGAAACCACAGT-3′	
Ese-14	(AGA)8	F-5′-GGGCGATCTTCAGGAACCAA-3′	254
		R-5′-GGAAGACCTCGCAACCGTTA-3′	
Ese-15	(ATC)8	F-5′-ACCAATCCTTCTGCCCAACA-3′	282
		R-5′-CTGCTGCTACCGCCATTTTC-3′	
Ese-20	(TGG)8	F-5′-CATGGTGGACGCGGAAATTC-3′	154
		R-5′-GCCATGAAGCAGAAGAGGGT-3′	
Ese-22	(GAT)8	F-5′-GCGTGTTGAAGAAGCTGCAA-3′	184
		R-5′-CACAGGCAATTCCTCCCGAT-3′	
Ese-24	(TTC)7	F-5′-CAGACTCCAACACCAACCGA-3′	190
		R-5′-CGGTGGATCGAAGAGAAGCA-3′	
Ese-32	(AAC)7	F-5′-CACCACGCAGCACTACCTAA-3′	240
		R-5′-TCGGCAGAAGTCGTCATGAT-3′	
Ese-55	(ATC)7	F-5′-TGCCAAAAGAAGCCGAACAA-3′	230
		R-5′-GGTTGCGGAGGAGAGAGAAG-3′	
Ese-65	(AGA)7	F-5′-CACCGAACAGAGAGTGAGGT-3′	232
		R-5′-AGATGCGGGGACTGGAAATG-3′	
Ese-72	(CTC)6	F-5′-TCCCCTTTTCCATCCTCTCT-3′	229
		R-5′-GAGACGACGATTGCCGAGAT-3′	
Ese-74	(GAA)6	F-5′-CGAAGAAGGGCAGCTTGAGA-3′	247
		R-5′-TGGACAGCGTATAAGCCCAT-3′	

## Data Availability

Data is contained within the article.
